# Defective mitochondrial protease LonP1 can cause classical mitochondrial disease

**DOI:** 10.1093/hmg/ddy080

**Published:** 2018-03-06

**Authors:** Bradley Peter, Christie L Waddington, Monika Oláhová, Ewen W Sommerville, Sila Hopton, Angela Pyle, Michael Champion, Monica Ohlson, Triinu Siibak, Zofia M A Chrzanowska-Lightowlers, Robert W Taylor, Maria Falkenberg, Robert N Lightowlers

**Affiliations:** 1 Institute of Biomedicine, Sahlgrenska Academy, University of Gothenburg, Gothenburg, Sweden; 2 Wellcome Centre for Mitochondrial Research, Institute of Neuroscience, The Medical School, Newcastle University, Newcastle upon Tyne, UK; 3 Department of Molecular and Human Genetics, Baylor College of Medicine, Houston, TX, USA; 4 Wellcome Centre for Mitochondrial Research, Institute of Genetics, The Medical School, Newcastle University, Newcastle upon Tyne, UK; 5 Department of Inherited Metabolic Disease, Guy's and St Thomas' NHS Foundation Trusts, Evelina London Children's Hospital, London, UK; 6 Wellcome Centre for Mitochondrial Research, Institute for Cell and Molecular Biosciences, The Medical School, Newcastle University, Newcastle upon Tyne, UK

## Abstract

LonP1 is a mitochondrial matrix protease whose selective substrate specificity is essential for maintaining mitochondrial homeostasis. Recessively inherited, pathogenic defects in LonP1 have been previously reported to underlie cerebral, ocular, dental, auricular and skeletal anomalies (CODAS) syndrome, a complex multisystemic and developmental disorder. Intriguingly, although classical mitochondrial disease presentations are well-known to exhibit marked clinical heterogeneity, the skeletal and dental features associated with CODAS syndrome are pathognomonic. We have applied whole exome sequencing to a patient with congenital lactic acidosis, muscle weakness, profound deficiencies in mitochondrial oxidative phosphorylation associated with loss of mtDNA copy number and MRI abnormalities consistent with Leigh syndrome, identifying biallelic variants in the *LONP1* (NM_004793.3) gene; c.1693T > C predicting p.(Tyr565His) and c.2197G > A predicting p.(Glu733Lys); no evidence of the classical skeletal or dental defects observed in CODAS syndrome patients were noted in our patient. *In vitro* experiments confirmed the p.(Tyr565His) LonP1 mutant alone could not bind or degrade a substrate, consistent with the predicted function of Tyr565, whilst a second missense [p.(Glu733Lys)] variant had minimal effect. Mixtures of p.(Tyr565His) mutant and wild-type LonP1 retained partial protease activity but this was severely depleted when the p.(Tyr565His) mutant was mixed with the p.(Glu733Lys) mutant, data consistent with the compound heterozygosity detected in our patient. In summary, we conclude that pathogenic *LONP1* variants can lead to a classical mitochondrial disease presentations associated with severe biochemical defects in oxidative phosphorylation in clinically relevant tissues.

## Introduction

LonP1, the mitochondrial lon protease homologue and member of the highly conserved AAA+ superfamily, is one of a small group of soluble proteases in the mitochondrial matrix (1,2). On import, the human protein is cleaved to form an approximately 100 kDa monomer that multimerizes to generate a ring-like, functional hexamer. It has a substrate selectivity, cleaving a variety of mitochondrial polypeptides and has been implicated in proteostasis, degrading unfolded and oxidatively damaged proteins (3). Further, roles as a direct binder of DNA or RNA have also been reported (4–6). Its exact physiological role may not be fully elucidated, but it is clear that it plays a vital function, since a homozygous deletion of LonP1 causes early embryonic lethality in mice (7). One of its natural substrates is the mitochondrial DNA (mtDNA) binding and packaging protein TFAM (8), which also has a crucial role in transcription initiation and mtDNA replication (9,10). LonP1 is therefore believed to play an essential role in mitochondrial gene expression and homeostasis (8).

Recent reports implicate pathogenic, recessively inherited *LONP1* variants in human disease phenotypes; two papers identified 11 patients with a profound and complex developmental disorder termed CODAS (or cerebral, ocular, dental, auricular and skeletal anomalies) syndrome (https://www.omim.org/entry/600373; date last accessed March 13, 2018) (11,12). Patients have a wide variety of clinical defects, some of which are common to mitochondrial disorders, such as hypotonia, short stature, brain atrophy and intellectual disability. However, there are marked skeletal abnormalities that are not commonly associated with classic mitochondrial disease presentation which are pathognomonic for CODAS syndrome.

Here we report the finding of compound heterozygous missense *LONP1* substitutions [c.1693T > C p.(Tyr565His) and c.2197G > A p.(Glu733Lys)] in a patient with classical mitochondrial disease manifestations including profound multiple OXPHOS deficiencies associated with the quantitative loss of mtDNA copy number in muscle. *In vitro* assessment of engineered LonP1 mutants revealed that the p.(Tyr565His) mutant had only a minimal effect on multimerization or ATP hydrolysis but was dramatically inhibited for binding or degrading TFAM, an effect that was partially suppressed by mixing with wild-type LonP1 protein. The second mutant, p.(Glu733Lys) had no marked effect alone on enzyme activity, but was unable to restore function when mixed with p.(Tyr565His) mutant protein. As segregation studies were not possible, it cannot be formally excluded that one of these mutations had occurred as a *de novo* event. However, our data are entirely consistent with the recessive inheritance of these variants and document for the first time that defective LonP1 can lead to a clinical disorder more classically associated with mitochondrial respiratory chain disease.

## Results

### Case report

The male proband was the second child of non-consanguineous parents born at 40 weeks gestation in good condition; birth weight 3490 g. At 24 h of age he presented with respiratory distress, poor feeding and a mixed acidosis with pH 6.85, pCO_2_ 6.7 kPa, bicarbonate 4.0 mmol/l, base deficit −25.9 and plasma lactate peaking at 21 mmol/l (normal range, 0.7–2.1 mmol/l). The metabolic acidosis was managed with intravenous bicarbonate and thiamine and biotin supplementation was introduced. He was noted to have an apnoea and possible seizure. Subsequent blood gases improved and enteral feeds were introduced. He was transferred to the Regional Metabolic Centre at 7 days of age for further investigation of the raised lactate.

Examination revealed no dysmorphic features nor organomegaly. Testes were undescended. The chest X-ray was normal with no bony abnormalities and echocardiogram revealed a structurally and functionally normal heart. Brain MRI showed a structurally normal brain with mildly increased white matter signal and MRS showed a lactate peak consistent with the hyperlactataemia; median 6.3 mmol/l (range, 1.5–14.0 mmol/l) (Supplementary Material, Fig. S1). A ketogenic diet was introduced with subsequent improvement (median 2.2 mmol/l; range 1.4–10.2 mmol/l). CPAP support was replaced with supplemental oxygen via nasal cannula. Episodes of respiratory distress continued and he remained nasogastric tube fed. Initial tone was normal with good antigravity power, but paucity of movement of the upper limbs and reduced active head movements developed whilst on the unit. Muscle and skin biopsies were taken for investigation of the congenital lactic acidosis. His prognosis remained guarded with frequent marked apnoeas.

Following discharge, his clinical condition gradually stabilized with less respiratory concerns and cessation of his marked apnoeas. The breathing pattern remained variable with periods of tachypnoea and bradypnoea. At 5 months of age, on review, a venous gas revealed chronic respiratory insufficiency with compensatory bicarbonate 31.4 mmol/l (normal range, 17–25 mmol/l). Growth centiles at that time were 50th centile for length and below the second centile for weight. There was some developmental progress, smiling at three months of age and developing hand regard. He would babble happily but remained tube fed. He died aged 7 months during an intercurrent upper respiratory infection.

### A diagnostic muscle biopsy reveals multiple OXPHOS deficiencies associated with severe mtDNA depletion

**Figure 1.**
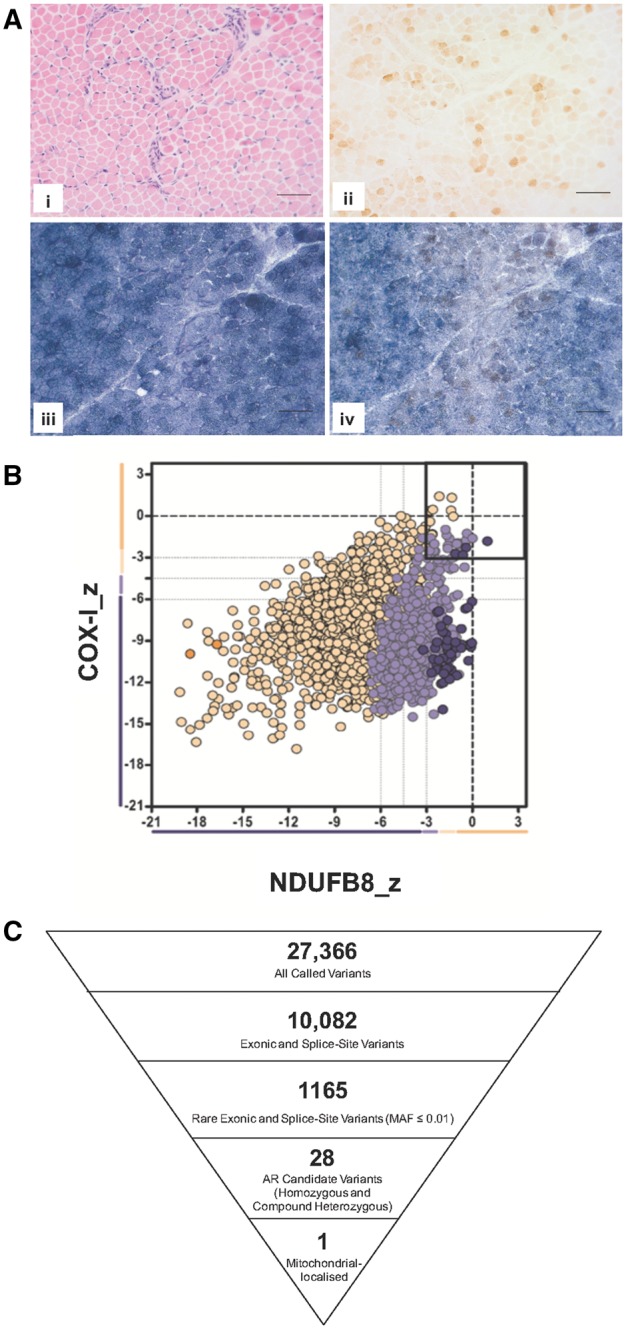
Muscle histopathology and identification of compound heterozygous *LONP1* variants. (**A**) Histochemical analysis of muscle from LonP1 patient. Serial transverse sections (10 μm) from a muscle biopsy from the patient were subjected to (i) haematoxylin and eosin staining and activity staining for (ii) cytochrome c oxidase (COX) and (iii) succinate dehydrogenase (SDH). (iv) Shows COX: SDH dual staining. Scale bar represents 50 μm. (**B**) Immunofluorescent analysis of individual muscle fibres from the LonP1 patient. Skeletal muscle sections (20 μm), were subjected to quadruple immunofluorescence as described (13). Signals were measured in individual muscle fibres from antibodies to NDUFB8 (complex I), COX1 (complex IV) and normalized to mitochondrial mass (porin). Each dot represents an individual muscle fibre, colour coded to represent mitochondrial mass (very low = dark purple; low = light purple; normal = yellow; high = orange). Images were used in conjunction with IMARIS software to quantify the levels of COXI and NDUFB8 in individual fibres as described. (**C**) WES analysis and filtering for rare, autosomal recessive variants in nuclear genes encoding mitochondrial-localized proteins identified only rare heterozygous *LONP1* variants.

A diagnostic muscle biopsy was evaluated using a range of histopathological and biochemical assays on suspicion of suspected mitochondrial disease. Haematoxylin and Eosin (H&E) staining of transverse muscle sections revealed areas of degenerating muscle with inflammatory infiltrate characterized by purple staining of nuclei clearly evident (Fig. 1Ai). The oxidative enzyme reaction for cytochrome *c* oxidase (COX) activity was weak throughout the section (Fig. 1Aii), in contrast to a relatively strong succinate dehydrogenase reactivity (Fig. 1Aiii). Sequential COX-SDH histochemistry confirmed a generalized decrease in COX reactivity throughout the section (Fig. 1Aiv). The spectrophotometric assessment of mitochondrial respiratory chain complex activities in another diagnostic centre revealed low activities of complexes I and IV (normalized to citrate synthase activity) (data not shown). The low levels of complex I and IV activities were further supported by quadruple immunofluorescence assays which use antibodies against porin (mitochondrial mass), NDUFB8 (complex I) and COX1 (complex IV) to determine relative levels of these complexes in individual fibres across the biopsy cryosection (Fig. 1B) (13).

This severe defect, suggestive of a generalized translation problem, was associated with severe depletion of mtDNA in patient muscle (<10% of age-matched controls; Supplementary Material, Fig. S2). Candidate screening of *SLC25A4* (NM_001151), *MPV17* (NM_002437), *SUCLG1* (NM_003849), *SUCLA2* (NM_003850), *TK2* (NM_004614) and *RRM2B* (NM_015713) was negative for pathogenic or likely pathogenic variants, therefore prompting WES using muscle DNA.

### Identification of rare compound heterozygous LONP1 variants

WES analysis for autosomal recessive variants in nuclear genes encoding mitochondrial-targeted proteins prioritized only two heterozygous missense variants in *LONP1* (NM_004793.3); c.1693T > C, p.(Tyr565His) (rs144125085) and c.2197G > A, p.(Glu733Lys) (Fig. 1C). Both variants were absent from in-house controls (*n* = 378), however they were represented in heterozygous form in ExAC (Exome Aggregation Consortium, http://exac.broadinstitute.org/). The c.1693T > C, p.(Tyr565His) variant was present in 1/118492 non-Finnish allele (MAF = 8.43 × 10^−6^), while the c.2197G > C, p.(Glu733Lys) variant was present in 2/119658 alleles (MAF = 1.671 × 10^−5^), a South Asian and a non-Finnish European. Segregation studies were not possible. Hence, to confirm that the two variants were present on different alleles, amplicons were generated that spanned both variants, cloned and eight clones were sequenced; four contained c.1693T > C, p.(Tyr565His) alone and four contained c.2197G > C, p.(Glu733Lys) alone (Supplementary Material, Fig. S3).

**Figure 2.**
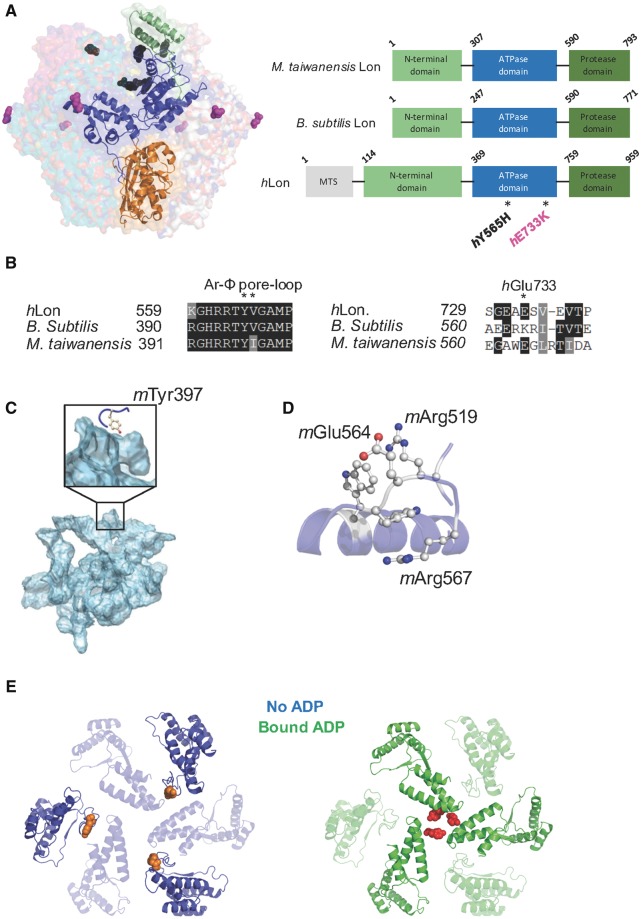
Structure analysis of human and bacterial Lon variants. (**A**) Domain organization of *H. sapiens*, *B. subtilis* and *M. taiwanensis* Lon. The structure of one monomer of the hexameric *M. taiwanensis* Lon is shown (PDB ID: 4YPL). The N-terminal domain is shown in green, AAA+ ATPase domain in blue and protease domain in orange. All three variants show similar domain organization, with the human variant containing an additional mitochondrial targeting sequence (MTS, grey). The predicted location of the two variants in human Lon, *h*Tyr565His and *h*Glu733Lys, are shown as black and magenta spheres, respectively. (**B**) Sequence alignments of human and bacterial Lon are shown around the two variants, Tyr565 and Glu733. (**C**) Analysis of internal channels of *M. taiwanensis* Lon revealed a large internal cavity leading from the substrate binding site to the protease domain. The *M. taiwanensis h*Tyr565 equivalent, *m*Tyr397, is located at the entrance to this channel and protrudes directly into the cavity. (**D**) The bacterial *h*Glu733 equivalent, *m*Glu564, is located on a loop in close proximity to *m*Arg519 and *m*Trp558. This may allow for a stabilizing salt-bridge interaction. (**E**) Model to illustrate the large conformational change that occurs on ADP binding. Large structural differences are observed in ADP-free (blue) and ADP-bound (green) subunits of *M. taiwanensis* Lon (PDB ID: 4YPL). The bacterial Tyr565 equivalent, *m*Tyr397, forms part of the conserved Ar-Φ pore-loop (Tyr-Val/Ile) implicated in substrate binding and translocation. The tyrosine residues are shown as orange (ADP-free state) or red (ADP-bound state) spheres in the top-down view.

LonP1 harbours an N-terminal mitochondrial presequence that is cleaved on entry to the mitochondrial matrix. The mature protein has three domains: a weakly conserved N-terminal domain, the AAA+ domain where ATP is bound and hydrolyzed and the C-terminal protease or P domain **(**Fig. 2A). The Tyr565 residue is highly conserved (comparisons shown between human, *Bacillus subtilis* and *Meiothermus taiwanensis* Lon proteases; Fig. 2B), occurs in the AAA+ ATPase domain and the variant was predicted to be damaging to protein function (Polyphen 2; Align GVGD; SIFT). AAA+ unfoldases, of which LonP1 is a member, are strongly evolutionarily conserved. One particular element termed the pore loop-1, protrudes into the central, substrate binding core of the hexamer from each subunit (14). A dipeptide aromatic-hydrophobic (Ar-Φ) motif is present within this element. In the ClpX protease, this motif is known to initially bind to the substrate and be involved in transducing energy from ATP hydrolysis to mechanical movement, effectively gripping and translocating the substrate as it is unfolded (15,16). In human LonP1, Tyr565 is that aromatic ortholog within the Ar-Φ pore-loop (Fig. 2B). There is no atomic resolution structure of the human LonP1 available. However, the *M. taiwanensis* structure is known and is well conserved (Supplementary Material, Fig. S4). The equivalent residue in *M. taiwanensis*, *m*Tyr397 is shown in (Fig. 2C). The region around *h*Glu733 is less well conserved, present near the boundary between the AAA+ ATPase and protease C-terminal domain and is not predicted to affect protein function (Fig. 2B and D). The internal pore ringed by mTyr397 residues constricts substantially on ADP binding, illustrating this area is subject to large conformational changes (Fig. 2E).

### A profound OXPHOS defect is present in muscle and fibroblasts from the patient

**Figure 3.**
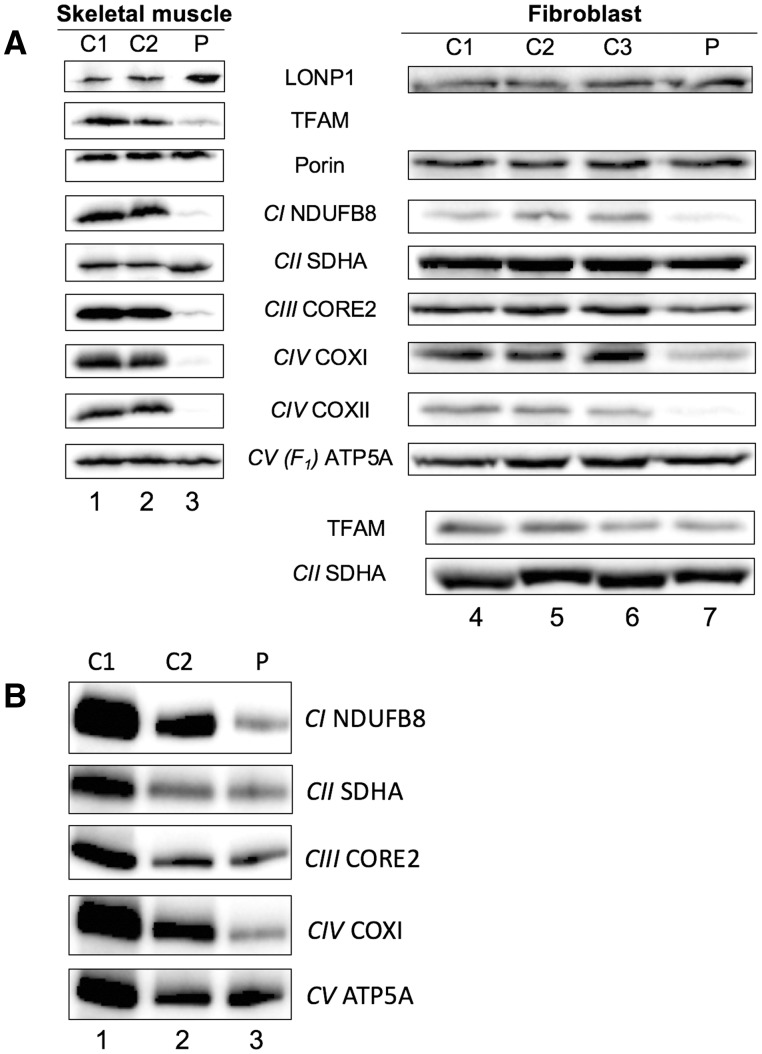
OXPHOS proteins are markedly depleted in LonP1 patient skeletal muscle and fibroblasts. (**A**) Western blot analysis of skeletal muscle and fibroblast extracts in patient and controls. Lysates (skeletal muscle, 30 μg; fibroblast, 40 μg) from different controls (skeletal muscle, lanes 1, 2; fibroblasts, lane 4, 5, 6) or patient (skeletal muscle, lane 3; fibroblasts, lane 7) were subjected to western blot analysis with antibodies to the indicated proteins as described. The relevant OXPHOS complex is shown in italics. TFAM is shown for fibroblasts lysates together with loading control derived from a separate blot. (**B**) BN-PAGE analysis of fibroblast extracts (100 μg) from two separate controls (lane 1, 2) or patient (lane 3) was performed as detailed. Each complex was visualized by the subunit-specific antibody indicated.

Skeletal muscle lysates from the patient and controls were prepared and subjected to gel electrophoresis to assess steady-state levels of core OXPHOS proteins. As shown in Figure 3, a profound decrease in markers for complexes I (NDUFB8), III (CORE2) and IV (COXI, II) was apparent, whilst the complex II marker (SDHA) was unaffected. These data are in accord with the decreased biochemical activities noted for complex I and complex IV and the immunohistochemical profile (Fig. 1B). Additionally, the major mitochondrial transcription and replication factor, TFAM, was also depleted in patient muscle, consistent with the observation of severe mtDNA depletion. LonP1 levels were mildly increased in the patient sample. A fibroblast cell line from the patient was established and extract analysed by both non-denaturing blue native and denaturing gel electrophoresis (Fig. 3A and B). Substantial decreases were shown for intact complex I and IV levels and a decrease in the steady-state level of NDUFB8, COXI and COXII were also apparent. Levels of TFAM in fibroblasts were unaffected, although contrary to the skeletal muscle sample the patient cell line had a >2-fold increase in mtDNA copy number (patient, 1060 ± 17 *n* = 6; control 1, 569 ± 15 *n* = 6; control 2, 417 ± 26; *n* = 5).

### Neither LONP1 mutant markedly affects oligomer formation

**Figure 4.**
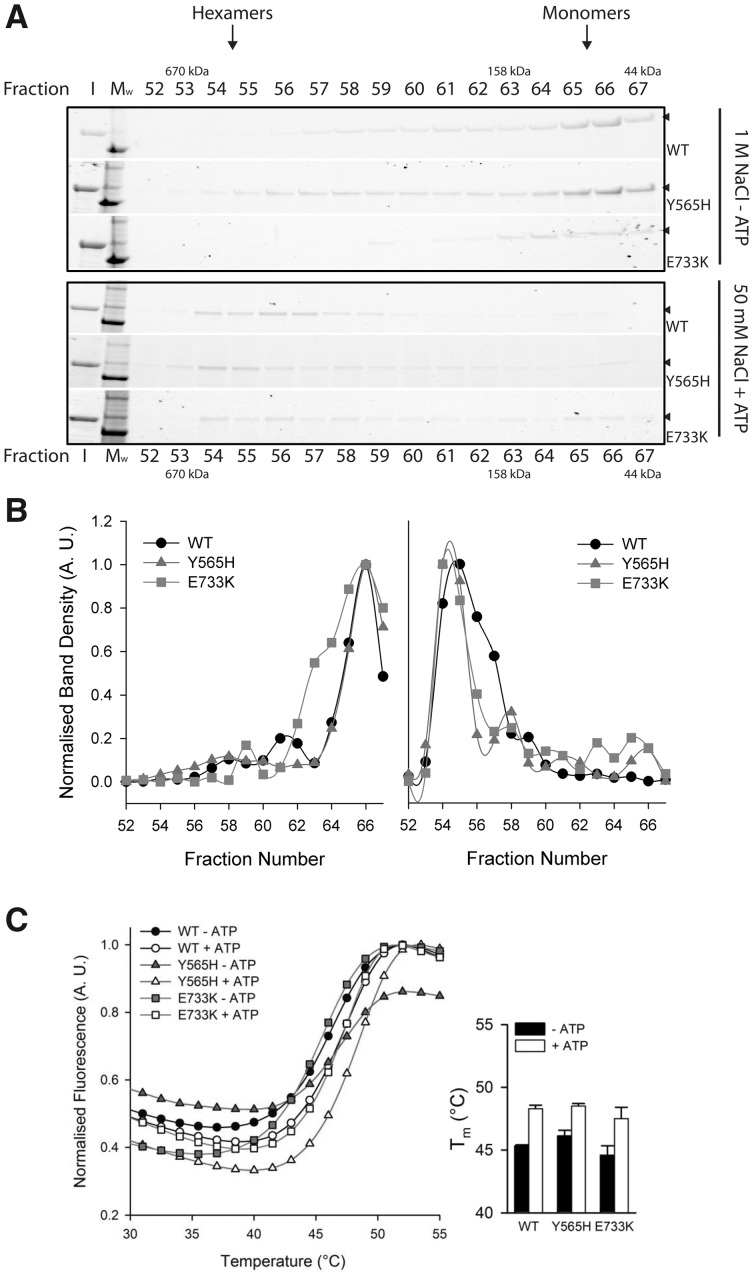
Multimerization comparisons and scanning fluorimetry. (**A**) The oligomeric states of LonP1 wild-type, p.(Tyr565His) and p.(Glu733Lys) were separated on a gel filtration column and resolved by SDS-PAGE. Samples were analysed in low salt (50 mm NaCl) and high salt (1 m NaCl) conditions without or with 4 mm ATP, respectively. The hexameric and monomeric fractions are indicated based on a size calibration curve. I, input; M_w_, molecular size marker. (**B**) Densitometric analysis of the fractions shown in (A) with 1 m NaCl and without 4 mm ATP (left panel) or 50 mm NaCl supplemented with 4 mm ATP (right panel). **(C)** Mutation-induced loss of LonP1 activity is not associated with global structural defects. Melting of various LonP1 forms **(**1.6 µm**)** was measured by differential scanning fluorometry in the presence of sypro orange ±4 mm ATP as described in Materials and Methods. Melting temperatures (*T*_m_), were calculated from the first derivative of fluorescence intensity against time and are shown for each LonP1 form.

To determine pathogenicity of both *LONP1* variants we attempted to reconstitute the mutant LonP1 forms *in vitro* and assess the effects of the variants on LonP1 functions. Mature proteins containing either the p.(Tyr565His) or p.(Glu733Lys) substitutions and harbouring a short C-terminal His tag to facilitate purification were expressed and purified along with a similar wild-type version. First, each of the LonP1 forms was assessed for their ability to form oligomers by comparing their mobility against defined molecular weight standards on a gel filtration column. As shown in Figure 4, high salt (1 m NaCl) and absence of ATP cofactor largely prevented the formation of higher order oligomer (Fig. 4A, top three panels; Fig. 4B, left panel), irrespective of whichever form of LonP1 was assessed. However, at low salt concentration and in the presence of ATP, multimers were favoured for all LonP1 forms (Fig. 4a, bottom three panels; Fig. 4B, right panel).

To confirm that either of the two variants did not cause any global structural defects, each oligomer was subjected to differential scanning fluorimetry in the presence and absence of 4 mm ATP (Fig. 4C). A modest ATP-induced stabilization was observed (Δ*T*_m_ ∼2–3°C) for all complexes, although no significant difference was seen between wild-type and mutant LonP1 proteins.

### The p.(Tyr565His) LONP1 mutant ablates proteolytic activity

**Figure 5.**
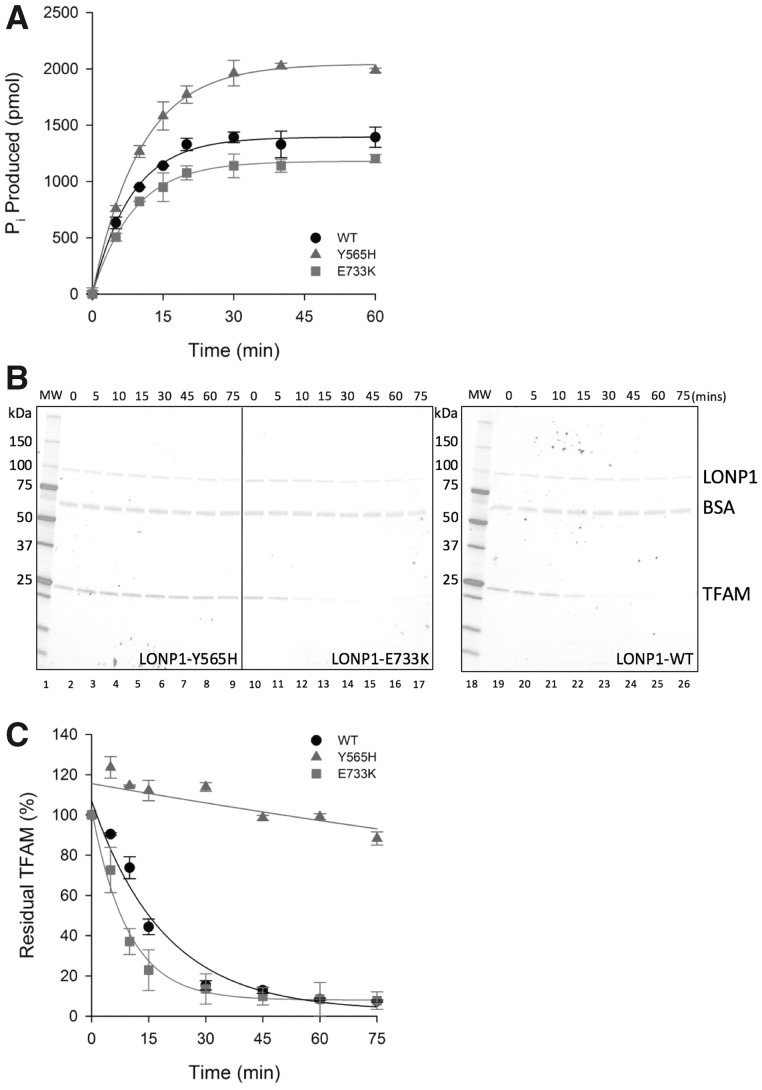
The p.(Tyr565His) mutant hydrolyzes ATP efficiently but is massively impaired in proteolytic activity. (**A)** ATP hydrolysis assays. The three LonP1 forms (2 μg) were measured for ATP hydrolytic activity during a 60 min time course as described. Data were fit to an exponential growth to maximum from which the pseudo first-order rate constant was calculated. **(B)** Proteolysis assays. The LonP1 substrate, TFAM, was incubated in the presence of 2 mm ATP supplemented with BSA and with either of the two LonP1 mutants (left panels) or wild-type (right panel). Aliquots were taken over a 75 min time course and separated through a precast 4–20% denaturing acrylamide gradient gel before visualization and densitometry. (**C)** Data from (B) were fit to an exponential decay using least squares non-linear regression analysis.

The rates of ATP hydrolysis of the three LonP1 forms were assessed using the Malachite Green Phosphate assay. As shown in Figure 5A and Table 1, rates were not significantly different between the three forms although total ATPase activity was higher for the p.(Tyr565His) mutant. The addition of the LonP1 protein substrate TFAM did not measurably affect ATP hydrolysis rates of any of the three forms (data not shown). Next, to determine whether the variants caused any modulation of protease activity, degradation assays were performed over a 75 min time course with TFAM as substrate. On ATP addition, strong proteolytic activity was noted for the wild-type and p.(Glu733Lys) forms (Fig. 5B and C and Table 1). However, there was negligible activity for p.(Tyr565His). Thus, p.(Tyr565His) is essentially catalytically inactive despite being capable of ATP hydrolysis.

**Table 1.** Catalytic activity of wild-type and mutant LonP1

LonP1 form	**ATP hydrolysis**^a^		**TFAM degradation**^b^	
	Maximal activity (pmol Pi formed)	Rate constant (min^−1^)	**TFAM remaining (%)**^c^	Rate constant (min^−1^)
WT	1394.8 ± 24.2	0.12 ± 0.008	12.9 ± 1.5	0.05 ± 0.01
Y565H	2043.9 ± 20.4	0.15 ± 0.005	99.1 ± 1.5	0.003 ± 0.001
E733K	1179.7 ± 12.6	0.11 ± 0.004	9.9 ± 4.3	0.11 ± 0.01
WT + Y565H	ND	ND	42.9 ± 3.1	0.02 ± 0.003
WT + E733K	ND	ND	20.8 ± 2.3	0.03 ± 0.003
Y565H + E733K	ND	ND	75.6 ± 0.4	0.009 ± 0.001

ND, not determined.

a Data fit to: *y* = a*(1 − exp(−b*x)), where *a* is the maximum value of *y* and *b* is the apparent rate constant.

b Data fit to: *y* = *y*_0_ + a*exp(−b*x), where *y*_0_ is the value of *y* at time 0, *a* is the minimum value of *y* and *b* is the apparent rate constant.

c Data shown are the % TFAM remaining after 45 min incubation compared with the 0 min control.

### The p.(Tyr565His) LONP1 mutant prevents binding to TFAM substrate

**Figure 6.**
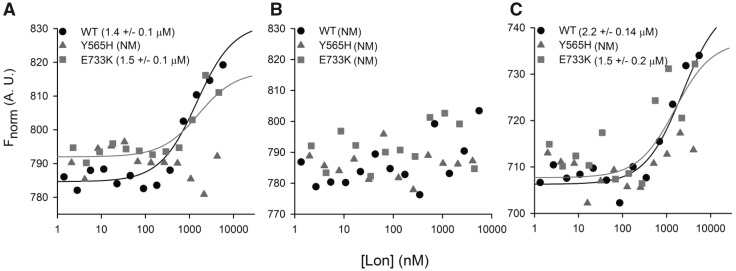
The p.(Tyr565His) mutant displays severe substrate binding defects. The binding interaction between LonP1 (4.65 µm) and its TFAM substrate (100 nm) was analysed by microscale thermophoresis as described above in buffer (**A**), 4 mm ATP (**B**) or 4 mm AMP-PNP (**C**). The equilibrium dissociation constant, *K*_d_ (μm) is given for each binding pair in parenthesis. NM: not measurable.

Previous reports have suggested that by mutating the Ar-Φ pore-loop in certain AAA+ family proteins, substrate binding was attenuated (14,15,17–19). To assess binding of TFAM to each of the LonP1 forms, TFAM was fluorescently labelled and microscale thermophoresis performed in the presence of ATP or the non-hydrolyzable ATP analogue AMP-PMP (Fig. 6). Low micromolar range binding affinities were apparent between TFAM substrate and either wild-type (1.36 ±0.09 μm) or p.(Glu733Lys) (1.52 ±0.1 μm) in buffer. An interaction between TFAM and p.(Tyr565His) LonP1 could not be detected under any of the conditions tested. The addition of ATP (Fig. 6B) resulted in substrate degradation and subsequent loss of fluorescent signal for wild-type and p.(Glu733Lys) LonP1 which made assessment of binding affinity impossible. Surprisingly AMP-PNP had little effect, marginally weakening the affinity for the two binders. This suggests that TFAM can interact with wild-type and p.(Glu733Lys) LonP1 without the necessity for ATP hydrolysis.

### Mixed mutant oligomers are severely attenuated for substrate binding and proteolysis

**Figure 7.**
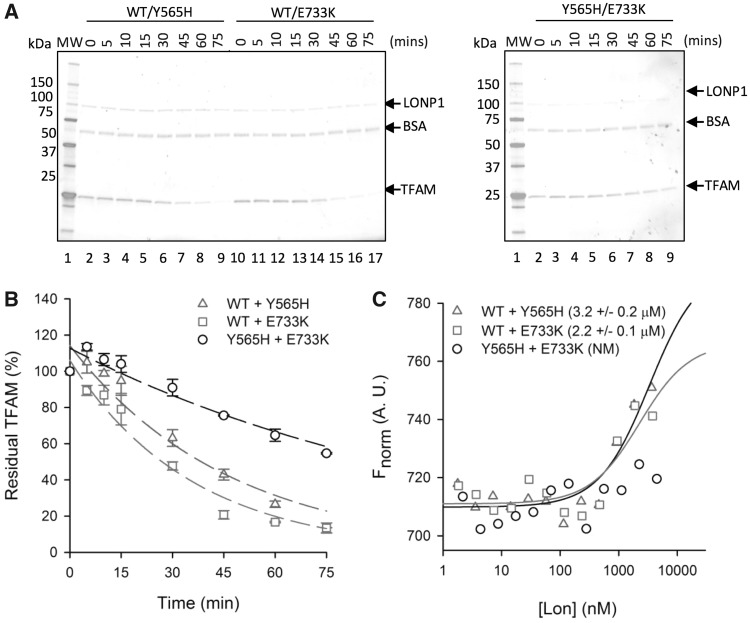
Mixed LonP1 mutant hexamers have severe TFAM binding and proteolytic defects. Hexameric mixtures (1: 1) of all LonP1 forms were prepared by dialysing the monomers from high salt as described above. (**A**) TFAM degradation assays were performed as described in the legend to Figure 4 with mixtures of wild-type and either of the two mutant forms (left panels) or with a mixture of both wild-type monomers (right). Amounts of remaining intact TFAM were calculated and fit to an exponential decay using least squares non-linear regression analysis (**B**). In (**C**), the binding between the mixtures of LonP1 and TFAM substrate in the presence of AMP-PNP were estimated by microscale thermophoresis. The equilibrium dissociation constant, *K*_d_ (μm) is given for each binding pair in parenthesis. NM; not measurable.

Data confirms the p.(Tyr565His) LonP1 mutant is unable to bind a model substrate, TFAM, and is consequently catalytically attenuated. By contrast, evidence to date suggested that the p.(Glu733Lys) mutant may have been a neutral polymorphism, as there was no appreciable effect on hexamer formation and the homohexamer was efficient in TFAM binding and proteolysis. Nevertheless, our *in vitro* experiments had yet to recapitulate the physiological situation for either parent or the proband, as genetic evidence indicated a mix of two forms of LonP1 in all cases: mutant and wild-type allele for each unaffected parent and the two mutant forms for the patient. To produce the mixed oligomers, equimolar amounts of various permutations of two LonP1 forms were added together in high salt to produce monomers before oligomerization was induced by dialysis. Following dialysis, aliquots of each mixture were assessed by gel filtration to confirm successful oligomerization and homo-oligomers were also reformed for WT **(**Supplementary Material, Fig. S5) and p.(Glu733Lys) to confirm the process did not itself cause loss of activity (Supplementary Material, Fig. S6). Mixtures were then subjected to TFAM-binding and proteolysis assays performed as detailed above (Fig. 7). When wild-type and p.(Glu733Lys) forms were mixed, binding was slightly weakened (WT 1.36 ± 0.09 μm*cf.* WT/E733K 2.17 ± 0.12 μm), consistent with a partial reduction in TFAM degradation activity (13% remaining TFAM after 45 min *cf*. 21% for WT/E733K; Table 1). When wild-type and the catalytically inactive p.(Tyr565His) mutant were mixed, TFAM-binding affinity was reduced (3.22 ± 0.17 μm). Unlike the p.(Tyr565His) mutant alone, however, protease activity was detected albeit reduced from WT (13% remaining TFAM after 45 min *cf.* 43% for WT/Y565H; Table 1). Both sets of data suggest this lower level of LonP1 activity was sufficient to assure an absence of clinical presentation for either parent who had a wild-type allele in tandem with either of the mutant LonP1 alleles. Interestingly, although the p.(Glu733Lys) LonP1 homohexamer had shown no defect in activity, it was partially affected when mixed together with the wild-type LonP1 (Fig. 7A and B). When mixed with the catalytically inactive p.(Tyr565His) mutant, a dramatic attenuation in both substrate binding and proteolysis was seen (Table 1). These data are consistent with the profound clinical phenotype in the patient being due to the loss of LonP1 activity and confirming pathogenicity of the compound heterozygous c.1693T > C p.(Tyr565His) and c.2197G > A p.(Glu733Lys) variants.

## Discussion

Recessively inherited *LONP1* variants have been previously linked to CODAS syndrome (https://www.omim.org/entry/600373), a unique disorder with distinctive clinical anomalies (11,12). Although the CODAS acronym signifies cerebral, ocular, dental, auricular and skeletal anomalies syndrome, reported patients appear to manifest a broad-spectrum of phenotypes ranging from isolated paediatric cataracts to severe CODAS-plus syndrome. This complex syndrome shares several clinical features of ‘classical’ mitochondrial disorders including intellectual disability, short stature, hypotonia and ptosis. However, there are skeletal and dental anomalies that are pathognomonic for CODAS syndrome. Recently, one individual presenting atypical CODAS syndrome with myopathy resembling Marinesco–Sjögren syndrome was described (20). Furthermore, a c.2014C > T, p.(Arg627Cys) *LONP1* variant has also been implicated in causing both CODAS syndrome and isolated paediatric cataracts (21).

By contrast, we identified rare biallelic variants in *LONP1*, c.1693T > C, p.(Tyr565His) and c.2197G > A, p.(Glu733Lys), in a patient presenting with congenital lactic acidosis, muscle weakness and brain MRI typical of Leigh syndrome, who had profound OXPHOS deficiencies and severe mtDNA depletion in skeletal muscle. Pathogenic *LONP1* variants have been reported at various positions throughout the protein, although most are typically located in the ATPase and proteolytic-domains. Several CODAS syndrome-linked variants are in close proximity to the identified c.2197G > A, p.(Glu733Lys) variant, including c.2161C > G, p.(Arg721Gly), c.2171C > T, p.(Ala724Val) and c.2245C > T, p.(Pro749Ser). Contrastingly, the c.1693T > C, p.(Tyr565His) variant also occurs in the ATPase domain but in the Ar-Φ pore-loop and is not proximal to other previously reported pathogenic variants. Taken together, the clinical presentation of our proband and *in vitro* studies suggest that the c.1693T > C, p.(Tyr565His) and c.2197G > A, p.(Glu733Lys) variants are unique. Thus, this supports an expanding clinical spectrum of *LONP1*-related disorders.

Our *in vitro* data provide compelling evidence that the c.1693T > C, p.(Tyr565His) variant causes profound defects in both LonP1 substrate binding and proteolytic activity. This highly conserved residue in the dipeptide domain of pore loop-1 has been found in numerous structural studies of similar AAA+ unfoldases/proteases to line the internal pore of the hexamer (15,22). It has been shown to interact directly with the substrate, and the coordinated concurrent large conformational changes around the ring of monomers, which occurs on ATP hydrolysis, results in translocation and unfolding of substrate that is necessary for proteolytic degradation (15). It is therefore entirely consistent with the biochemical data reported above that this variant is pathogenic. TFAM binding and proteolytic degradation were unmeasurable, whilst formation of hexamer was not significantly affected and the hexamer was as thermally stable as the wild-type LonP1. Mixing the wild-type and p.(Tyr565His) LonP1 monomers prior to hexamer formation resulted in weaker affinity for TFAM and a decreased rate of degradation but represented a substantial increase over p.(Tyr565His) alone. We cannot determine from our experiments whether this is due to mixed heterohexamers having reduced activity or whether the mutant somehow blocked mixed hexamer formation with the wild-type monomer, resulting mainly in two discrete populations of homohexamer. However, this weaker but clearly present activity was consistent with the recessive nature of the variant.

Predicting the effect of the second variant, c.2197G > A, p.(Glu733Lys), from structural data is more complex. The residue is not completely conserved and is found immediately N-terminal to a short beta sheet (b9) at the end of the AAA+ domain and prior to the protease domain linker. Pathogenic variants at this site have never previously been implicated in pathogenesis. This substitution in the homohexamer does not significantly reduce ATP hydrolysis, TFAM binding or proteolytic function *in vitro*. However, when p.(Glu733Lys) is mixed with wild-type LonP1, activity is diminished (Fig. 5B and C*cf*Fig. 7A and B) and when mixed with the p.(Tyr565His) mutant there is a profound decrease in activity. Clearly, the p.(Glu733Lys) mutant also has a negative impact on LonP1 activity but the negative effect is somehow rescued if all monomers in the hexamer contain the substitution. The reason for this remains unclear, although it is consistent with the genetics of the family. This may be attributed to local structural deformations around the mutated residue. For the p.(Glu733Lys) LonP1 mutant alone, the effects of this deformation may be neutralized due to its presence in all subunits of the homohexamer. However, when p.(Glu733Lys) is mixed with wild-type or p.(Tyr565His) LonP1, the resulting heterohexamers may no longer be capable of this rescue due to conformational differences between adjacent subunits. Whereas wild-type subunits would be able to partially rescue the activity of a Tyr565-containing p.(Glu733Lys) LonP1, the p.(Tyr565His) mutant—which cannot bind or translocate substrate—would not. This is consistent with observations in *E. coli* ClpX (15), where removal of even a few pore loop tyrosine residues lead to a loss of substrate binding.

Finally, we were able to observe that loss of LonP1 activity led to a massive decrease in respiratory chain complex activity in patient skeletal muscle. This is entirely consistent with the depletion of mtDNA, which encodes key components of these respiratory chain complexes. Why, however, does the absence of LonP1 activity lead to mtDNA depletion? The exact connection is unclear. LonP1 has been shown to regulate mtDNA copy number in *Drosophila melanogaster* by cleaving TFAM (8). It is known that TFAM and mtDNA have a mutual dependence for stability, whereby TFAM binds mtDNA and protects it from degradation, but when not bound to mtDNA, TFAM is rapidly degraded (9). Whilst LonP1-mediated TFAM degradation may have an acute role in regulating mtDNA levels by causing the loss of excess TFAM and thus reducing mtDNA levels, it cannot fully explain the observation in this patient. It has been demonstrated that small changes in TFAM levels dramatically impact the fraction of mtDNA molecules available for transcription and DNA replication. At high TFAM/DNA ratio TFAM forms stable protein filaments on DNA that block melting and prevent progression of the replication and transcription machineries (23). Therefore, failure to reduce TFAM levels may inhibit mtDNA replication and explain the mtDNA depletion seen in the patient. The mtDNA depletion itself would, in turn, explain the decrease in TFAM level. Further, LonP1 is not a non-specific protease but it has numerous known protein substrates in the mitochondrial matrix. It is highly likely that the absence of LonP1 activity will have many pleiotropic effects, one of which must result in the chronic loss of mtDNA content.

## Materials and Methods

### Whole exome sequencing, analysis and interpretation

Exome capture of fragmented genomic DNA from muscle was attained using the Illumina TruSeq Rapid Exome (45 Mb) capture kit, sequenced using the Illumina HiSeq 2000 platform in 100 bp reads and aligned to the human reference genome (UCSC hg38). Called variants were restricted to exonic (coding) or splice-site variants with a minor allele frequency (MAF) less than or equal to 0.01 (1%) from 378 in-house controls and external variant databases, including ExAC and the 1000 Genomes Project. Autosomal recessive (homozygous or compound heterozygous) variants in nuclear genes encoding mitochondrial-targeted proteins were prioritized. Pathogenicity of missense variants was assessed using Polyphen 2 (http://genetics.bwh.harvard.edu/pph2/), Align GVGD (http://agvgd.hci.utah.edu/) and SIFT (http://sift.jcvi.org/). Identified candidate variants were validated by Sanger sequencing.

### Histopathological and biochemical analyses of OXPHOS function

Routine histological and histochemical analyses of skeletal muscle cryosections (10 μm) were undertaken as part of the diagnostic work-up. The activities of the respiratory chain complexes and the matrix marker enzyme, citrate synthase, were determined as described previously (24). Quantitative immunofluorescence analysis of frozen skeletal muscle cryosections was undertaken to assess the levels of OXPHOS complexes I (anti-NDUFB8) and IV (anti-COXI) relative to a marker of mitochondrial content (anti-VDAC1) essentially as described (13).

### Cell culture

Primary paediatric control and *LONP1* patient fibroblasts were cultured in Eagle’s minimal essential medium (Sigma Aldrich) supplemented with 10% (v/v) fetal calf serum, 1× non-essential amino acids, 1 mm sodium pyruvate and 50 µg/ml uridine. Cell lines were propagated at 37°C in humidified 5% CO_2_.

### Protein extract production, western blot and blue native gel analyses

For total protein extraction from muscle, frozen age-matched control or patient muscle tissue (10 mg) was crushed in RIPA buffer [1% Igepal CA-630, 0.5% sodium deoxycholate, 0.1% EDTA, 10 mm β-mercaptoethanol, 10 µg/ml PMSF, 15 µl/ml Triton X-100, 1× EDTA-free protease inhibitor (Pierce)]. The homogenate was vortexed, incubated on ice and then homogenized with the T25 Ultra-Turrax homogenizer (IKA). Homogenates were centrifuged at 14 000*g*, 10 min, 4°C and the supernatants retained. For cultured cell extracts, fibroblasts were harvested and lysed in 50 mm Tris–HCl pH 7.5, 130 mm NaCl, 2 mm MgCl_2_, 1 mm PMSF, 1% NP-40 and 1× EDTA-free protease inhibitor cocktail (Pierce). Protein lysates were separated on 12% polyacrylamide gels using SDS-PAGE and transferred to a PVDF membrane via electrophoresis. The membranes were blocked in 5% milk/TBS-T and immunoblotted using primary and HRP-conjugated secondary antibodies. Extraction of mitochondrial membranes and Blue-native polyacrylamide-gel electrophoresis (BN-PAGE) analysis was performed according to previously described methodologies (25). Briefly, mitochondrial extracts were solubilized with *n*-dodecyl b-d-maltoside (Sigma) and 100 µg of control and patient samples were electrophoretically separated on a 4–16% native polyacrylamide BisTris gradient gel (Life Technologies). Complexes were transferred onto PVDF membranes and subjected to immunoblotting with primary antibodies: NDUFB8 (complex I), SDHA (complex II), UQCRC2 (complex III), COXI (complex IV), and ATP5A (complex V).

The following primary antibodies were sourced as follows: OXPHOS Human Cocktail (Abcam, ab110411), NDUFB8 (Abcam, ab110242), SDHA (Abcam, ab14715), CORE2 (Invitrogen, A11143), COXI (Abcam, ab14705), COXII (Abcam, ab110258), ATP5A (Life Technologies, 7F9BG1), LONP1 (Sigma, HOA002192), TFAM (Abcam, ab119684), VDAC1 (Abcam, ab14734) and β-actin (Sigma, A1978).

### Production of LonP1 forms, expression and purification

Wild-type LonP1 lacking the mitochondrial targeting sequence [1–114; (26)] was cloned into a pET-20b vector with a 6×X His tag at the C-terminus. LonP1 variants were generated using the QuickChange Lightning site-directed mutagenesis kit (Stratagene) according to the manufacturer’s instructions. Protein expression was induced with 1 mm IPTG at 37°C for 2.5 h. Cells were subsequently harvested, frozen in liquid nitrogen and thawed at 4°C in lysis buffer containing 25 mm Tris–HCl pH 8.0, 10 mm β-mercaptoethanol and 1× protease inhibitors. Cells were then lysed using an Ultra-Turrax T3 homogenizer (IKA) and supernatant was loaded onto a His-Select Nickel Affinity Gel (Sigma-Aldrich) equilibrated with 20 mm imidazole in buffer A (25 mm Tris–HCl, pH 8.0, 0.4 m NaCl, 10% glycerol, 10 mm β-mercaptoethanol and 1× protease inhibitors) followed by elution by 250 mm imidazole in buffer A. Proteins were subsequently purified over a 5 ml HiTrap Heparin column (GE Healthcare) and a 1 ml HiTrap MonoQ column (GE Healthcare). Both HiTrap columns were equilibrated in buffer B (20 mm Tris–HCl, pH 8.0, 0.1 m NaCl, 10% glycerol, 0.5 mm EDTA and 1 mm DTT) and the proteins were eluted by a salt gradient (0.2–1.2 m NaCl in buffer B). Lastly, proteins were purified over a HiLoad 16/60 Superdex 200 gel filtration column (GE Healthcare) equilibrated in buffer C (20 mm Tris–HCl, pH 8.0, 0.4 m NaCl, 10% glycerol and 2 mm DTT). The purity of the protein was estimated by SDS-polyacrylamide gel electrophoresis and Coomassie Brilliant Blue staining.

### Microscale thermophoresis

TFAM was cysteine labelled using a 2-fold excess of NT-547-maleimide dye according to the manufacturer’s instructions. Twelve two-fold dilutions of unlabelled LonP1 or 1: 1 mixtures of WT/p.(Tyr565His), WT/p.(Glu733Lys) or p.(Tyr565His)/p.(Glu733Lys) (2.3–4650 nm) were made in MST buffer (50 mm Tris–HCl, pH 8.0, 10 mm MgCl_2_, 10% glycerol) supplemented with 4 mm ATP or AMP-PNP. Dilutions were incubated with 200 nm labelled TFAM for 10 min at 24°C. Samples were analysed in triplicate using a Monolith NT.115 instrument (NanoTemper Technologies, Munich, Germany) with standard capillaries, 80% LED power and 60% MST power for 30 s at 24°C. Thermophoresis data were baseline corrected and used to calculate *K*_d_, bound state and unbound state in the NanoTemper analysis software. The standard error was calculated from three independent measurements.

### Oligomerization assay/gel filtration analysis

Hexamerization of wild-type, p.(Tyr565His) and p.(Glu733Lys) LonP1 was monitored by gel-filtration chromatography using a Superose 6 Increase 10/300 column (GE Healthcare) pre-equilibrated in running buffer A (20 mm Tris–HCl, pH 8.0, 10% glycerol, 2 mm DTT, 1 m NaCl) or B (20 mm Tris–HCl, pH 8.0, 10% glycerol, 2 mm DTT, 50 mm NaCl and 10 mm MgCl_2_). Each protein was dialyzed into running buffer A or B with buffer B samples incubated at 37°C for 10 min in the presence of 1 mm ATP. Samples (200 µl) were injected onto the column with a flow rate of 200 µl/min and monitored by absorbance at 280 nm. Fractions of 250 µl were collected, analysed by SDS-PAGE and bands quantified using ImageLab software (BioRad). A size calibration curve was prepared using thyroglobulin (670 kDa), γ-globulin (158 kDa), ovalbumin (44 kDa), myoglobin (17 kDa) and vitamin B12 (1.35 kDa) according to the manufacturer’s instructions (BioRad). All samples were analysed in triplicate and representative gels shown.

### Thermofluor stability assay

The fluorescent dye Sypro Orange (Invitrogen) was used to monitor the temperature-induced unfolding of wild-type and mutant LonP1. Wild-type and mutant proteins (1.6 µm final) were set up in 96 well PCR plates in assay buffer (50 mm Tris–HCl, pH 8 and 50 mm NaCl) in the absence or presence of 4 mm ATP. Differential scanning fluorimetry was performed in a C1000 Thermal Cycler using the CFX96 real time software (BioRad). Scans were recorded using the HEX emission filter (560–580 nm) between 4 and 95°C in 0.5°C increments with a 5 s equilibration time. The melting temperature (*T*_m_) was determined from the first derivative of a plot of fluorescence intensity versus temperature (27). The standard error was calculated from three independent measurements.

#### Protein structure modelling

The structure of the *Meiothermus taiwanensis* LonA protease (PDB ID: 4YPL) was used to model the effects of the human p.(Tyr565His) and p.(Glu733Lys) variants in PyMol (The PyMol Molecular Graphics System, Version 2.0, Schrödinger, LLC). The equivalent *M. taiwanensis* residues were identified by sequence alignment of the human and bacterial Lon proteins in Clustal Omega (https://www.ebi.ac.uk/Tools/msa/clustalo/; date last accessed March 13, 2018). Analysis of internal cavities was performed using the 3V server with default settings (28).

### ATP hydrolysis assay

*In vitro* ATPase assays were performed using the Malachite Green Phosphate Assay kit (BioAssay systems, POMG-25H) according to manufacturer’s instructions. Briefly, samples were prepared ± ATP with 2 µg recombinant LonP1 in a total volume of 20 µl per reaction. A standard curve (0–40 µm phosphate) was also prepared in triplicate. The samples were incubated at 37°C for 0–60 min and the reaction stopped by addition of 80 µl working reagent. Samples were loaded onto Nunclan 96-well flat transparent plates and incubated at room temperature for 25 min followed by measurement A_630_ nm on the Tecan Infinite M200 using Magellan 6 software. Standard error was calculated from three independent measurements.

### TFAM proteolysis assay

*In vitro* TFAM proteolysis assays were performed in 10 µl reactions with 9.5 pmol recombinant LonP1 protein, ±6.5 pmol TFAM and 2× buffer (100 mm NaCl, 50 mm Tris–HCl, pH 8, 10 mm MgCl_2_, 0.1 mg/ml BSA) with or without 2 mm ATP. Recombinant LonP1 and TFAM proteins were diluted in 2× buffer without ATP if necessary. The samples were incubated at 37°C for 0–75 min, respectively and then immediately placed on ice. Samples were separated on a precast 4–20% gradient SDS-PAGE gel (BioRad, 567–8094) and visualized on a ChemiDoc MP Imager using ImageLab™ (BioRad) to determine the efficiency of TFAM degradation.

## Supplementary Material

Supplementary Material is available at *HMG* online.

## Supplementary Material

Supplementary Figures
