# Supplementary figures and images for: Defective mitochondrial protease LonP1 can cause classical mitochondrial disease
Source: Hum Mol Genet. 2018 Mar 6;27(10):1743–53. doi: 10.1093/hmg/ddy080 (PMC5932559; doi:10.1093/hmg/ddy080)

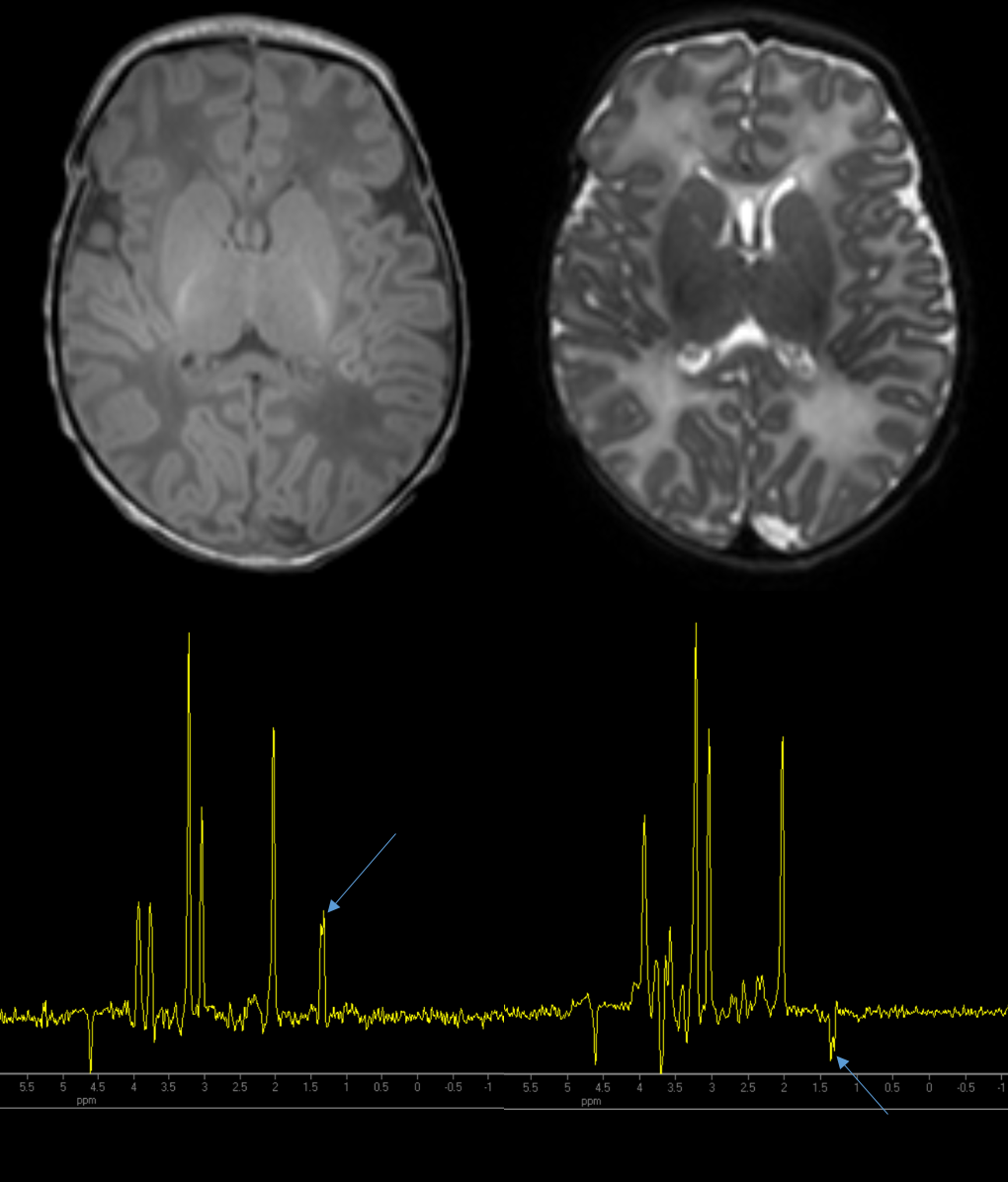

Supplement: Supplementary Figures [file ddy080_suppl_figures.zip › LonP1 Peter et al. New Supp Fig1.tif]

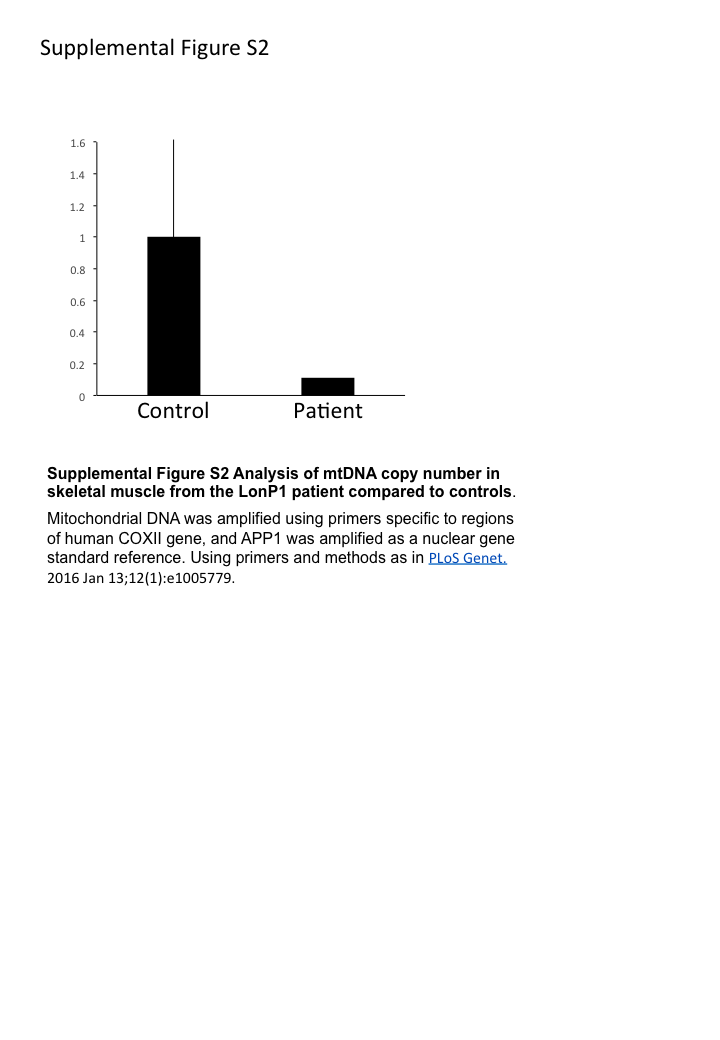

Supplement: Supplementary Figures [file ddy080_suppl_figures.zip › LonP1 Peter et al. New Supp Fig2.tiff]

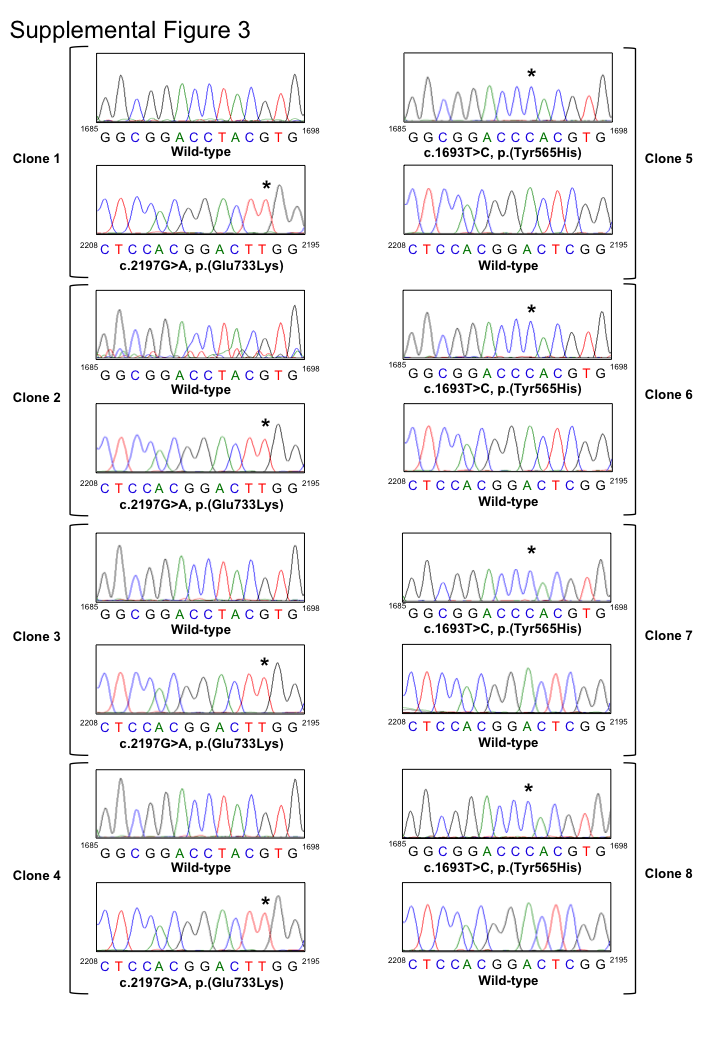

Supplement: Supplementary Figures [file ddy080_suppl_figures.zip › LonP1 Peter et al. New Supp Fig3.tiff]

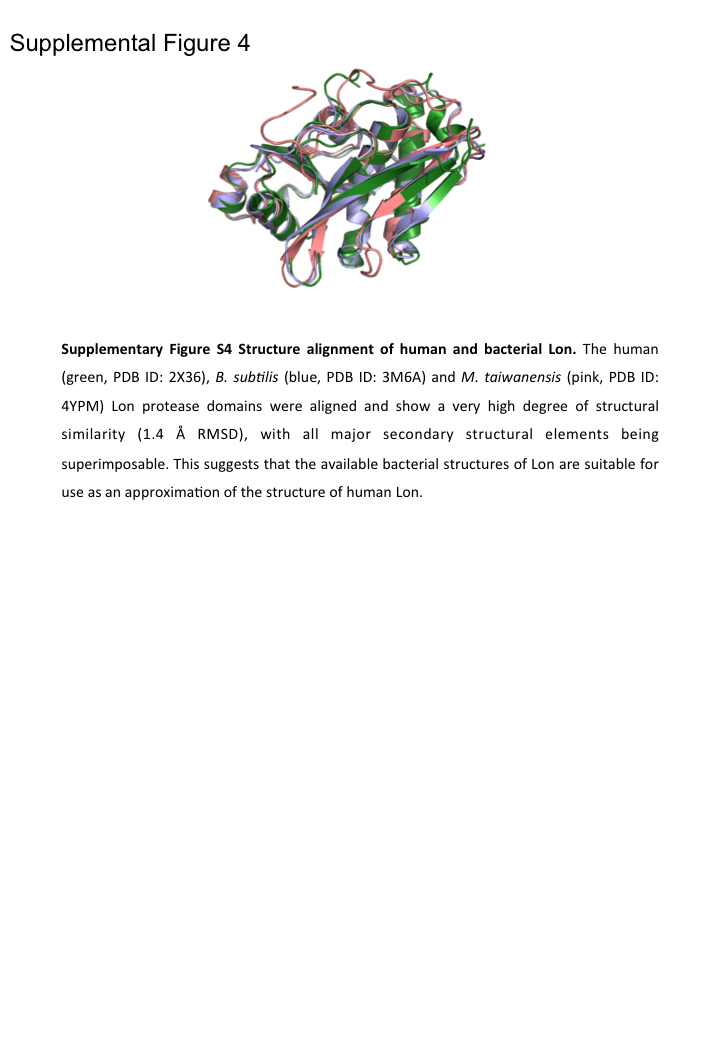

Supplement: Supplementary Figures [file ddy080_suppl_figures.zip › LonP1 Peter et al. New Supp Fig4.tiff]

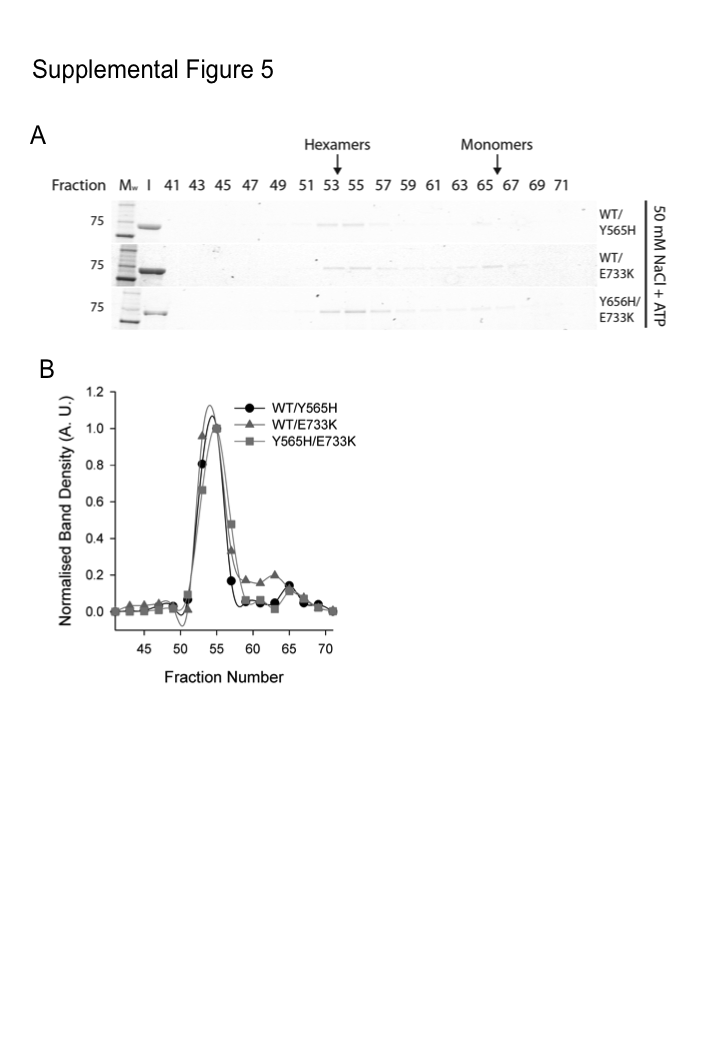

Supplement: Supplementary Figures [file ddy080_suppl_figures.zip › LonP1 Peter et al. New Supp Fig5.tiff]

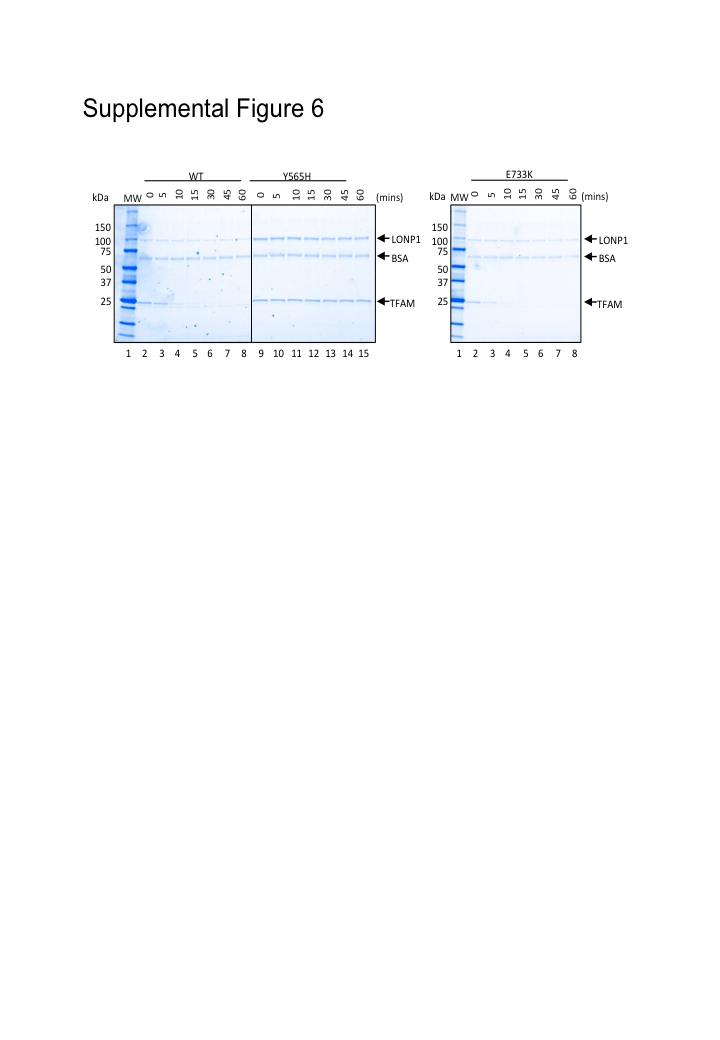

Supplement: Supplementary Figures [file ddy080_suppl_figures.zip › LonP1 Peter et al. New Supp Fig6.tiff]
